# Bacterial Colony Counts During Vaginal Surgery

**DOI:** 10.1080/10647440300025515

**Published:** 2003

**Authors:** Patrick Culligan, Michael Heit, Linda Blackwell, Miles Murphy, Carol A. Graham, James Snyder

**Affiliations:** 1 Division of Urogynecology and Reconstructive Pelvic Surgery Department of Obstetrics Gynecology and Women’s Health 315 East Broadway M-18 Louisville KY 40202 USA; 2 Division of Laboratory Medicine Department of Pathology University of Louisville Health Sciences Center Louisville KY USA

## Abstract

*Objective:* To describe the bacterial types and colony counts present before and during vaginal surgery.

*Methods:* A descriptive study was undertaken of patients undergoing vaginal hysterectomy with or without reconstructive pelvic surgery. Aerobic and anaerobic bacterial cultures were obtained immediately before and throughout the surgical cases at preselected time intervals. Standard antimicrobial prophylaxis was administered in all cases. Mean total colony counts and mean anaerobic colony counts were determined by adding all colonies regardless of bacteria type. ‘Contamination’ was defined as ≥ 5000 colony-forming units/ml.

*Results:* A total of 31 patients aged 26 to 82 years (mean age ± SD, 51 ± 15) were included. The highest total and anaerobic colony counts were found at the first intraoperative time interval. On the first set of cultures (30 minutes after the surgical scrub), 52% (16/31) of the surgical fields were contaminated, and at 90 minutes, 41% (12/29) were contaminated. A negligible number of subsequent cultures were contaminated.

*Conclusions:* Any future interventions designed to minimize bacterial colony counts should focus on the first 30 to 90 minutes of surgery.


Infect Dis Obstet Gynecol 2003;11:161–165

### Bacterial colony counts during vaginal surgery

**Patrick Culligan[1], Michael Heit[1], Linda Blackwell[1], Miles Murphy[1], Carol A. Graham[1] and James Snyder[2]**

[1]*Division of Urogynecology and Reconstructive Pelvic Surgery, Department of Obstetrics, Gynecology and Women's Health, and* [2]*Division of Laboratory Medicine, Department of Pathology, University of Louisville Health Sciences Center, Louisville, KY*

***Objective:*** To describe the bacterial types and colony counts present before and during vaginal surgery.
***Methods:*** A descriptive study was undertaken of patients undergoing vaginal hysterectomy with or without reconstructive pelvic surgery. Aerobic and anaerobic bacterial cultures were obtained immediately before and throughout the surgical cases at preselected time intervals. Standard antimicrobial prophylaxis was administered in all cases. Mean total colony counts and mean anaerobic colony counts were determined by adding all colonies regardless of bacteria type. 'Contamination' was defined as ≥ 5000 colony-forming units/ml.
***Results:*** A total of 31 patients aged 26 to 82 years (mean age ± SD, 51 ± 15) were included. The highest total and anaerobic colony counts were found at the first intraoperative time interval. On the first set of cultures (30 minutes after the surgical scrub), 52% (16/31) of the surgical fields were contaminated, and at 90 minutes, 41% (12/29) were contaminated. A negligible number of subsequent cultures were contaminated.
***Conclusions:*** Any future interventions designed to minimize bacterial colony counts should focus on the first 30 to 90 minutes of surgery.

Key words: POSTOPERATIVE INFECTION; VAGINAL HYSTERECTOMY

Post-hysterectomy wound infections primarily result from the ascending spread of micro-organisms from the upper vagina[1]. Prior to the widespread use of prophylactic antibiotics, the rate of wound infection after vaginal hysterectomy was around 30–40%[2]. This unacceptably high infection rate prompted at least 25 randomized controlled trials and two meta-analyses[3,4], all of which supported the use of prophylactic antibiotics to decrease the infectious morbidity rate and length of hospital stay associated with vaginal hysterectomy. As a result, the American College of Obstetricians and Gynecologists currently recommends the use of antimicrobial prophylaxis for all patients undergoing vaginal hysterectomy[5]. The current rate of operative site infection after vaginal hysterectomy has declined to between 2.1% and 9.5%[6].

Nevertheless, infection remains the most common complication associated with vaginal hysterectomy[7]. Of the nearly 600 000 hysterectomies that are performed in the USA each year, about 150 000 (25%) are performed vaginally[8]. Using the above figures (i.e. multiplying the infection rate[6] by the number of vaginal hysterectomies[7]), the number of wound infections following vaginal hysterectomy in the USA may be estimated to be between 3150 and 14 250 per year.

An ideal strategy for continuing to lower infection rates after vaginal hysterectomy would be to conduct randomized controlled trials using

Correspondence to: Patrick Culligan, MD, 315 East Broadway M-18, Louisville, KY 40202, USA. Email: pculligan@louisville.edu

© 2003 The Parthenon Publishing Group 

operative site infection as the primary outcome measure. However, such studies would require large numbers of patients in order to detect a clinically significant difference in postoperative infections. For example, a randomized controlled trial with 80% power for detecting a reduction in infection rate from 6% to 3% would require 814 patients in each arm. Such a study would be very difficult and expensive to complete.

Use of a surrogate end point as the outcome measure might allow researchers to design more feasible studies. Bacterial colony counts in the vaginal field would be a reasonable surrogate end point for randomized controlled trials, because operative site infections following vaginal hysterectomy are thought to result from direct contamination by vaginal flora[7]. Any intervention that reduces the bacterial colony counts present in the operative field might therefore reduce operative site infection rates.

However, before this surrogate end point could be used in a randomized controlled trial, we need to know the baseline bacterial colony counts present throughout typical vaginal surgery. To date, this information has not been reported. Therefore, the objective of the present study was to describe the bacterial types and colony counts present before and during vaginal surgery. Our specific aim was to generate pilot data for a randomized controlled interventional trial with the goal of reducing bacterial colony counts present in the operative field during vaginal surgery.

### MATERIALS AND METHODS

This was a descriptive study approved by the University of Louisville Health Sciences Center Human Studies Committee. Between September 2001 and April 2002, all patients undergoing vaginal hysterectomy with or without pelvic reconstructive surgery in our center were offered enrollment. No patients refused enrollment.

Standard infection prophylaxis, including preoperative intravenous antibiotics and a 5-minute surgical scrub with povidone–iodine, was used for all patients. Preoperative antibiotics were administered between 30 minutes and 2 hours prior to the start of each operation. Cefazolin (1 g) was used unless an allergy to this medication was reported. The alternative antibiotic regimen consisted of 900 mg of clindamycin and 120 mg of gentamicin.

Immediately prior to administration of the preoperative antibiotics, baseline aerobic and anaerobic cultures of vaginal flora were obtained. A standard technique was used to collect these cultures. All cultures for this study were obtained using a combined aerobic/anaerobic collection and transport system (CultureSwab™ Plus, Becton Dickenson, Franklin Lakes, NJ, USA). With the patient in the dorsal lithotomy position, a swab was placed in the posterior vaginal fornix and agitated throughout the length and circumference of the vagina in a standard fashion for 1 minute. Care was taken to include the entire surface area of the vagina, but the cervix was avoided. We chose not to obtain cultures via vaginal washings because of the inherent difficulty of retrieving fluid from the operative site during vaginal surgery.

A similar standardized technique was used to obtain cultures of the vaginal field 30 minutes after completion of the surgical scrub and hourly thereafter throughout each patient's surgery. Exact time intervals between cultures were determined with a stopwatch. When it was time for a given culture, the surgeon would swab the entire vaginal field, avoiding the cervix or peritoneal cavity.

Immediately after each operation, the culture transport tubes were taken to the University of Louisville Hospital Microbiology Laboratory for processing. All swabs submitted for culture were placed in 1 ml of sterile saline and vortexed. A sterile, calibrated (0.01 ml) loop was used to inoculate the specimen on to 5% sheep blood agar and chocolate agar plates which were incubated at 35°C in 5–10% carbon dioxide (aerobic cultures). Cultures for anaerobic microorganisms were inoculated quantitatively on brucella blood agar (BBA), phenylethyl alcohol agar (PEA), kanamycin vancomycin agar (KV), and *Bacteroides* bile esculin agar (BBE). Manual colony counts were reported for all positive cultures, with identification performed according to standard biochemical methods. The approach to quantifying microbial flora involved a 0.01-ml calibrated loop. Using this technique, one colony is equivalent to 100 colony-forming units/ml of specimen. A total colony count was determined, and this was followed by observing and then counting each specific colony type. We do not routinely screen patients for bacterial vaginosis prior to vaginal surgery. None of the patients in this group were screened for bacterial vaginosis because none of them presented with clinical findings consistent with that disorder.

Our end point of interest was defined in two ways, each in relation to the timing of the various cultures taken before and during surgery. First, we defined our end point as a continuous variable by calculating the mean total and anaerobic colony counts at each designated time interval. Mean total colony counts were calculated by adding all colonies of bacteria found at a given time interval regardless of species, and then dividing by the number of patients in question at each time interval. Mean total anaerobic colony counts were determined at each time interval in a similar fashion.

Secondly, we defined our end point as a categorical variable by labeling the vaginal field 'contaminated' if total bacterial colony counts were ≥ 5000 colony-forming units/ml. This cut-off value for contamination was chosen because it correlates with a reading of 1-plus on simple dip-stick analysis. We also followed the postoperative course of each patient in the study group to identify any operative site infections.

Descriptive statistical analysis for the group was performed using SPSS version 11.0 (SPSS Inc., Chicago, IL, USA). No group comparisons were performed because this was a simple pilot study.

### RESULTS

A total of 31 patients ranging in age from 26 to 82 years (mean ± SD, 51 ± 15 years) were included. Operative times ranged from 25 to 270 minutes (average time, 156 ± 61 minutes). A total of 26 patients received cephazolin prophylaxis, and the remaining 5 patients received clindamycin and gentamicin. In addition to vaginal hysterectomy, 29 patients underwent reconstructive pelvic surgery, including 29 vaginal vault suspensions, 25 rectocele repairs, 10 bilateral salpingo-oophorectomies and 23 tension-free vaginal-tape suburethral slings. The remaining two patients had a vaginal hysterectomy alone. The average parity in the group was 3.26 ± 2.0, and the average body mass index was 29.3 ± 7.7 kg/m$^2$. In total, 25 patients (81%) in the study group were Caucasian, and the remaining six patients (19%) were African-American; Fifteen patients (48%) in the group were postmenopausal, and four of these women were taking combined oral hormone replacement therapy at the time of surgery.

Baseline cultures revealed normal vaginal flora in all cases (Table 1). None of the baseline cultures were negative and, as expected, a wide variety of aerobic and anaerobic pathogens was found.

Figure 1 shows that the highest total colony counts were found at the first intraoperative time interval (i.e. 30 minutes after completion of the surgical scrub). Anaerobic bacteria were equally suppressed throughout all culture groups (Figure 2).

On the first set of cultures (i.e. 30 minutes after the surgical scrub), 52% (16/31) of patients had ≥ 5000 colonies of bacteria present. In total, 29 operations lasted long enough to result in a second set of cultures, and 41% (12/29) of these cultures revealed ≥ 5000 bacterial colonies. A third set of cultures (150 minutes after the surgical scrub) was collected in 24 patients, and 25% (6/24) of these

**Table 1** Bacteria found in baseline (preoperative) vaginal cultures

| *Bacteria type* | *Proportion of patients (n = 1) with given bacteria type on baseline culture* |
|---|---|
| Anaerobic pathogens[a] | 45% (14/31) |
| Coagulase-negative *Staphylococcus aureus* | 58% (18/31) |
| *Staphylococcus aureus* | 16% (5/31) |
| λ-Hemolytic streptococci | 10% (3/31) |
| α-Hemolytic streptococci | 23% (7/31) |
| *Enterococcus faecalis* | 10% (3/31) |
| *Escherichia coli* | 42% (13/31) |
| Gram-negative bacilli[b] | 13% (4/31) |
| Lactobacilli | 87% (27/31) |
| *Klebsiella pneumoniae* | 13% (4/31) |
| Group B streptococci | 13% (4/31) |
| Coryneforms | 13% (4/31) |

[a]Includes *Peptostreptococcus, Bacteroides, Clostridium perfringens* and *Prevotella melaninogenica*; [b]Includes *Alcaligenes, Enterobacter, Serratia* and *Proteus*

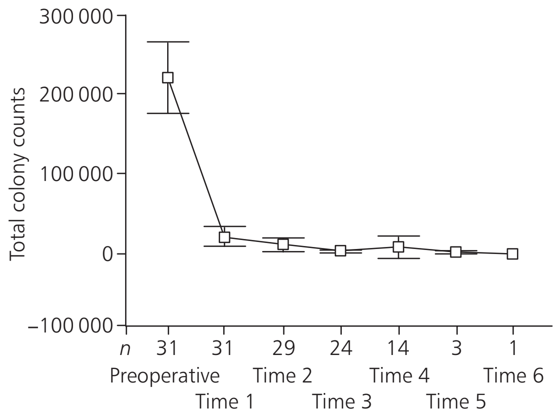

**Figure 1** Total colony counts including all bacteria as a function of intraoperative time. Error bars represent 95% confidence intervals. Time 1 = 30 minutes after povidone–iodine preparation. Time 2 = 1 hour after time 1. Each subsequent time is 1 hour after the previous one

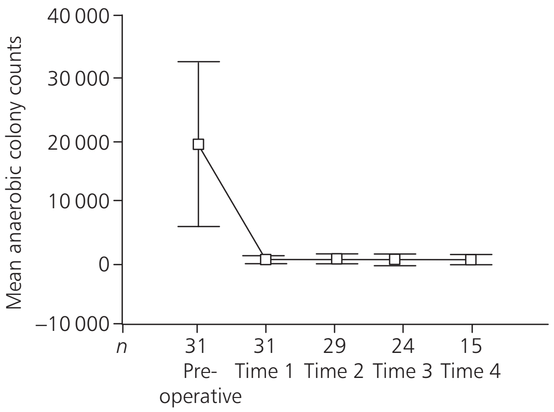

**Figure 2** Anaerobic bacterial colony counts as a function of intraoperative time. Error bars represent 95% confidence intervals. Time 1 = 30 minutes after povidone–iodine preparation. Time 2 = 1 hour after time 1. Each subsequent time is 1 hour after the previous one were contaminated. Of the 14 patients for whom a fourth set of cultures was obtained, only two (14%) were contaminated, and none of the subsequent cultures for any patient contained ≥ 5000 colonies.

At each intraoperative time interval, some of the patients' cultures were completely negative. This occurred in 29% (9/31) of the first set of cultures, 35% (10/29) of the second set, 42% (10/24) of the third set and 86% (12/14) of the fourth set.

None of the 31 patients developed operative site infections.

### DISCUSSION

All of the successful techniques for infection prophylaxis have one thing in common – they decrease the number of bacterial colony counts at the operative site. These techniques have worked so well for gynecologic cases that it is no longer feasible to use actual infections as the outcome measure for future studies of infection prophylaxis. Therefore a surrogate end point such as perioperative bacterial colony counts would be useful. Presumably any intervention that could decrease the number of bacteria present in the operative field would also reduce the rate of operative site infections.

This is the first description of the bacterial colony counts present in the operative field before and during vaginal surgery when standard infection prophylaxis is used. The strengths of this study include the relatively homogenous population and the standardization of specimen collection and processing.

Despite strict adherence to the aseptic protocol, the highest bacterial colony counts were found 30 to 90 minutes after the surgical scrub, possibly implicating the latter as the 'weakest link' of standard infection prophylaxis protocols. Thereafter, mean bacterial colony counts decreased sharply, possibly due to the preoperative antibiotics. Therefore any future interventions designed to minimize intraoperative bacterial colony counts should focus on the first 30 to 90 minutes of the operation. Perhaps the use of a different surgical scrub preparation and/or antibiotic regimen would be a more effective way to prepare the vaginal field for surgery, by further reducing colony counts during the first 90 minutes of surgery.

### ACKNOWLEDGEMENTS

This study was funded by an ACOG American College of Obstetricians and Gynecologists/3M research award for the study of infectious disease in women. The authors wish to thank the University of Louisville hospital microbiology staff for their technical support.

### REFERENCES

1. Hemsell DL. Gynecologic postoperative infections and antimicrobial prophylaxis. In Mandell GL, Bennett JE, Dolin R, eds. *Principles and Practice of Infectious Diseases*, 5th edn. Philadelphia, PA: Churchill Livingstone, 2000:3177–91
2. Bolling DR, Plunkett GD. Prophylactic antibiotics for vaginal hysterectomies. *Obstet Gynecol* 1973; 41:689–92
3. Tanos V, Rojansky N. Prophylactic antibiotics in abdominal hysterectomy. *J Am Coll Surg* 1994; 179:593–600
4. Mittendorf R, Aronson MP, Berry RE, *et al.* Avoiding serious infections associated with abdominal hysterectomy: a meta-analysis of antibiotic prophylaxis. *Am J Obstet Gynecol* 1993;169: 1119–24
5. American College of Obstetricians and Gynecologists. *Antibiotic Prophylaxis for Gynecologic Procedures*. ACOG Practice Bulletin Number 23. American College of Obstetricians and Gynecologists, 2001
6. Culver DH, Horan TC, Gaynes RP, *et al.* Surgical wound infection rates by wound class, operative procedure and patient risk index. *Am J Med* 1991; 91(Suppl. 3B):152–157
7. Duff P, Park RC. Antibiotic prophylaxis in vaginal hysterectomy. *Obstet Gynecol* 1980;55(Suppl. 5): 193–202
8. Farquhar CM, Steiner CA. Hysterectomy rates in the United States, 1990–1997. *Obstet Gynecol* 2002: 99;229–34

Received 02/20/03; Accepted 05/22/03
